# Advances in Zeroing Neural Networks: Bio-Inspired Structures, Performance Enhancements, and Applications

**DOI:** 10.3390/biomimetics10050279

**Published:** 2025-04-29

**Authors:** Yufei Wang, Cheng Hua, Ameer Hamza Khan

**Affiliations:** 1 College of Computer Science and Engineering, Jishou University, Jishou 416000, China; yufeiwang@stu.jsu.edu.cn (Y.W.); chenghua@jsu.edu.cn (C.H.); 2 Smart City Research Institute (SCRI), Hong Kong Polytechnic University, Kowloon, Hong Kong; 3 Department of Land Surveying and Geo-Informatics (LSGI), Hong Kong Polytechnic University, Kowloon, Hong Kong

**Keywords:** zeroing neural network (ZNN), noise-tolerant, time-varying problems, convergence, applications

## Abstract

Zeroing neural networks (ZNN), as a specialized class of bio-Iinspired neural networks, emulate the adaptive mechanisms of biological systems, allowing for continuous adjustments in response to external variations. Compared to traditional numerical methods and common neural networks (such as gradient-based and recurrent neural networks), this adaptive capability enables the ZNN to rapidly and accurately solve time-varying problems. By leveraging dynamic zeroing error functions, the ZNN exhibits distinct advantages in addressing complex time-varying challenges, including matrix inversion, nonlinear equation solving, and quadratic optimization. This paper provides a comprehensive review of the evolution of ZNN model formulations, with a particular focus on single-integral and double-integral structures. Additionally, we systematically examine existing nonlinear activation functions, which play a crucial role in determining the convergence speed and noise robustness of ZNN models. Finally, we explore the diverse applications of ZNN models across various domains, including robot path planning, motion control, multi-agent coordination, and chaotic system regulation.

## 1. Introduction

Neural networks, recognized as versatile and highly efficient computational models, have found extensive applications across diverse fields [1,2,3,4,5,6,7], especially in the modeling, prediction, and optimization of complex problems [4,8,9,10,11,12,13]. By mimicking the architecture and operational principles of biological neural systems, these networks are adept at uncovering hidden patterns within large datasets. Consequently, they have become indispensable tools in tasks such as decision support, pattern recognition, and numerous other data-driven applications [14,15,16,17,18].

The development of biomimetic neural networks has been profoundly influenced by the recurrent neural network (RNN) model proposed by Hopfield [19], a distinguished member of the U.S. National Academy of Sciences. His model represents neural networks as graph structures composed of nodes (neurons) and connections (weights), exerting a significant impact on the field of computational neuroscience. Each node corresponds to a neuron, while the connections encode interactions between neurons. Despite its relatively simple architecture, the Hopfield network exhibits remarkable dynamical properties, earning its recognition as one of the foundational models in neural network research.

Unlike traditional optimization algorithms that rely on gradient information for problem-solving, some biomimetic algorithms have been proposed to address non-convex optimization problems [20,21,22,23,24,25,26,27]. These include the Egret Swarm Optimization Algorithm [28,29,30], the Cuckoo Search Algorithm [31], the Harmony Search Algorithm [32], the Grey Wolf Optimizer and Multi-Strategy Optimization Methods [33], the Whale Optimization Algorithm [34], the Harris Hawks Algorithm (VEH) [35,36], and Ant Colony Optimization [37]. Based on the theoretical framework of the RNN, the zeroing neural network (ZNN) is a biologically inspired subclass of RNN systematically proposed by Zhang et al. in 2002 [38]. It aims to emulate the adaptive behavior of biological systems in response to external changes and is specifically designed for high-precision and robust solutions to optimization and time-varying problems. Unlike traditional RNNs that rely on energy function-based update mechanisms, the ZNN adopts a neurodynamic approach by constructing an error-monitoring function, enabling the system states to dynamically converge toward a zero-error trajectory. This mechanism, known as the “error-zeroing mechanism”, essentially mimics the homeostatic regulation process in biological neurons, where negative feedback continuously corrects deviations from the target, ensuring that the system state approaches a predefined zero-error point.

Compared with conventional methods for solving time-varying problems, such as sliding mode control, adaptive control, or numerical integrators, the ZNN demonstrates superior computational efficiency, while maintaining high precision and strong robustness. It organically integrates the adaptive nature of neural networks with the dynamic regulation strengths of control theory, exhibiting unique advantages in handling time-varying problems. As such, the ZNN occupies an irreplaceable and significant position within the neural network paradigm.

Initially, researchers applied the ZNN in the real-number domain to address the time-varying matrix inversion (TVMI) problem [39,40,41,42]. Unlike traditional neural networks (for example, gradient-based optimization techniques in neural networks, including gradient neural networks (GNN) and RNN ). The ZNN has also been applied to complex matrix inversion tasks, including time-varying complex matrix inversion (TVCMI) [43,44,45,46] and time-varying complex matrix pseudoinversion (TVCMP) [47,48]. In addition, the ZNN has also been cited for solving time-varying equations, such as time-varying overdetermined linear systems (TVOLS) [49], time-varying nonlinear equations (TVNE) [50,51], time-varying Stein matrix equations (TVSME), and the time-varying Sylvester matrix equation (TVSME2) [52,53]. In the field of optimization, the ZNN can also be applied to handle such problems, including time-varying nonlinear minimization (TVNM) [54], nonconvex nonlinear programming (NNP) [55,56,57,58], multi-objective optimization (MOO) [59], time-varying quadratic optimization (TVQO) [60,61,62,63,64], and time-varying nonlinear optimization (TVNO) [65,66].

The ZNN enhances its capacity to address time-varying problems primarily by modifying activation functions and integration methods [67]. Notable examples include the Li activation function [68], the FAESAF and FASSAF activation functions [69], the multiplication-based sigmoid activation function [70], the NF1 and NF2 activation functions [71], the symbolic quadratic activation function [72], as well as the hyperbolic sine activation function [73], and the multiply-accumulate activation function [74]. These functions significantly impact the network’s nonlinear characteristics and convergence rate. The ZNN can also be classified into single-integration and double-integration types based on the integration method. The single-integral ZNN [75] corrects errors by incorporating time derivatives, thereby enabling it to tackle relatively simple time-varying problems, such as motion planning and path tracking. In contrast, the double-integration ZNN incorporates the second-order derivative of the error [76], improving the system’s adaptability to highly dynamic and nonlinear problems. It is particularly effective for more complex time-varying control tasks, such as multi-agent systems and chaotic control [77]. By combining these activation functions and integration methods, the ZNN can also efficiently address dynamic time-varying problems through the flexible setting of both variable and fixed parameters [78]. In many practical applications, variable parameters empower the ZNN to adjust its output in real time, enabling it to adapt to dynamic system changes, while fixed parameters establish a stable framework for addressing static or nearly static problems. By carefully designing these parameters, the ZNN can effectively manage more complex and multidimensional time-varying systems [79].

Researchers have extended the application of the ZNN to several practical fields [80]. In robotics, the ZNN has been employed for robotic arm path tracking [81,82] and motion planning [83,84,85], enabling high-precision movements in complex environments through the real-time dynamic adjustment of control strategies [86,87]. Additionally, it has been utilized to optimize robotic paths for improved efficiency. In multi-agent systems, the ZNN facilitates the coordination of multiple agents [88,89], achieving group collaboration and synchronization, particularly in addressing global optimization challenges in complex tasks. In the field of chaotic control, the ZNN dynamically adjusts system parameters in real time to mitigate chaotic phenomena and ensure system stability. For signal processing, the ZNN has demonstrated effectiveness in noise suppression and signal recovery, particularly in image and speech processing, where it efficiently removes noise and restores the quality of original signals. Furthermore, the ZNN finds extensive applications in engineering optimization problems, such as in automatic control systems, where it supports the real-time adjustment and optimization of control strategies, ensuring stable and efficient system operation.

**Figure 1:**
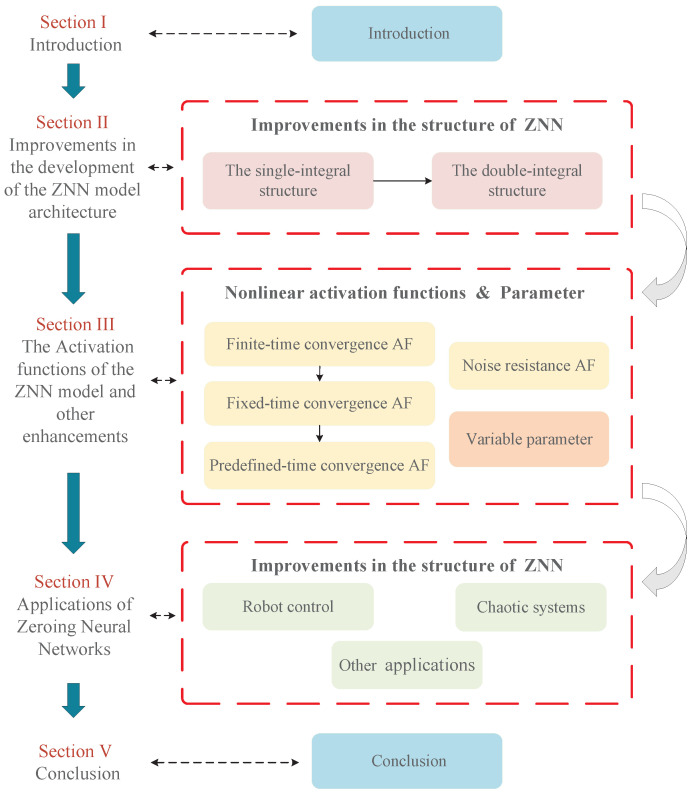
Block diagram of the structure in this paper.

Therefore, this paper presents a comprehensive review of the development of ZNN models and their diverse applications. The paper’s framework is illustrated in Figure 1, and the remaining sections are organized as follows:

Section 2 offers a detailed review of the structural development of the ZNN model. Section 3 examines nonlinear activation functions and time-varying parameters, with a focus on their roles in enhancing convergence and improving noise tolerance. Section 4 presents a summary of the practical applications of the ZNN in real-world domains. Section 5 concludes the paper, summarizing key findings and suggesting potential directions for future research.

## 2. Improvement of Zeroing Neural Network Model Structures

This section offers a comprehensive review of the advancements in ZNN models over the past decade, with a primary focus on the design of model structures. It highlights significant research achievements across diverse problem domains and establishes a robust theoretical and practical framework for further analysis.

### 2.1. Original Zeroing Neural Network Model

The gradient neural network (GNN) method was initially introduced by researchers to solve optimization problems [90]. The GNN is a type of neural network based on gradient optimization principles, specifically designed to address a wide range of optimization challenges and the resolution of dynamic system problems. The central concept behind the GNN involves constructing a performance index and optimizing it through gradient descent, thereby providing a solution to the problem at hand. A key feature of the GNN [91] is the definition of a scalar performance index J(x), which quantifies the deviation of the system state *x* from the desired target state. A common expression for the performance index is

$$J\left(x\right)=\frac{1}{2}{∥f\left(x\right)∥}^{2}.$$

Here, f(x) denotes the nonlinear function or constraint equation to be solved, typically framed as a root-finding problem where f(x)=0. The fundamental design equation of the GNN is expressed as

$$\dot{v}\left(t\right)=-\nabla \;J\left(x\right).$$

The GNN can be expressed as

$$\dot{v}\left(t\right)=-f{\left(x\right)}^{T}\nabla \;f\left(x\right).$$

As the scale of the problems increased, researchers observed that applying the GNN often resulted in significant residual errors. To address this limitation, Zhang et al. proposed the ZNN, specifically designed to accurately solve time-varying scientific computing problems. Since its introduction, the ZNN has been widely applied across various domains [92]. In contrast to the GNN, which relies on optimizing a performance index, the ZNN directly constructs an error function E(t), such as

(1) E(t)=B(t)Y(t)−I.

The primary objective of the ZNN is to regulate the network dynamics such that the E(t) asymptotically approaches zero over time.

The design equation of the ZNN is given by

(2) $$\dot{E}\left(t\right)=-\gamma E\left(t\right),$$

where the parameter γ governs the decay rate of the error function. A larger value can accelerate convergence but may increase sensitivity to noise and system stiffness, whereas a smaller value results in slower but smoother convergence. Moderately increasing γ can improve accuracy; however, it often requires a smaller step size, leading to increased computational cost. Therefore, γ should be carefully selected based on simulation requirements and available hardware resources.

In contrast to the traditional GNN, the ZNN offers significant advantages in addressing time-varying parameter problems. The ZNN tracks the solution trajectory of time-varying systems in real time by incorporating the time derivative of residual errors, leading to faster convergence and improved stability. It demonstrates strong robustness against noise and disturbances and eliminates the need for iterative weight updates, thereby reducing computational complexity and enhancing real-time adaptability. Unlike the objective function optimization approach employed by the GNN, the ZNN controls the system by minimizing errors and dynamically adjusting them to accommodate system changes. This enables the ZNN to excel in managing time-varying problems, precisely controlling errors, and effectively mitigating the vanishing or exploding gradient issues commonly encountered in the GNN. As a result, the ZNN is better suited for handling long-term dependencies and dynamic variations.

### 2.2. Integration-Enhanced Zeroing Neural Network

To enhance the model’s noise robustness, Jin et al. (2015) first proposed the integration enhanced zeroing neural network (IEZNN) [93], building upon the original ZNN. By incorporating a single integral term, the IEZNN improves the network’s stability, convergence, and ability to suppress noise while effectively handling time-varying systems. In the original ZNN, the error function is typically used to measure the deviation between the system’s output and the desired result. In contrast, the error function in the IEZNN not only depends on the current error but also integrates past errors, enabling smoother dynamic transitions. This approach mitigates the instability caused by instantaneous error fluctuations, particularly when addressing time-varying and uncertain problems. The IEZNN model controls the evolution of the error by incorporating the single integral term. The design equation can be expressed as

(3) $$\begin{array}{c} \dot{E}\left(t\right)=-\gamma E\left(t\right)-\lambda_{1}\int_{0}^{t}E\left(\tau \right)d\tau , \end{array}$$

where γ>0 and $\lambda_{1}>0$ are convergence parameters. This equation ensures that the error decreases progressively over time, eventually converging to zero. The IEZNN is an implicit dynamic system that considers not only the current state error but also integrates past error information. This approach enhances the system’s stability, especially when operating in time-varying environments. The inclusion of the single-integral term enhances the robustness of the IEZNN, particularly in the presence of noise and disturbances. By mitigating instantaneous error fluctuations, the IEZNN improves its ability to handle uncertainty and external disturbances, making it well-suited for real-time computation in dynamic environments. The network effectively tracks time-varying matrices and computes their values, ensuring smooth convergence based on matrix value errors. This capability is especially critical when noise interference is significant, as the IEZNN maintains high computational accuracy, particularly when solving noisy time-varying Lyapunov equations (TVLE) [94], Liao et al. combined nonlinear activation functions with integral terms to propose a unified design formula for the zeroing neural dynamics (ZND). Building on this formula, they introduced the bounded zeroing neural dynamics (BZND) model. First, the error function is defined as

$$E\left(t\right)=A{\left(t\right)}^{T}Z\left(t\right)+Z\left(t\right)A\left(t\right)+B\left(t\right).$$

The design formula for ZND is

(4) $$\begin{array}{c} \dot{E}\left(t\right)=-\lambda_{1}F_{1}\left(E\left(t\right)\right)-\gamma F_{2}\left(E\left(t\right)-\lambda_{1}\int_{0}^{t}F_{1}\left(E\left(\iota \right)\right)d\iota \right). \end{array}$$

Here, γ∈(0,+∞) and $\lambda_{1}\in \left(0,+\infty \right)$ are scaling factors that adjust the convergence rate. The nonlinear activation function arrays $F_{1}\left(\cdot \right)$ and $F_{1}\left(\cdot \right)$ play a pivotal role in the dynamic process of the model. Under noisy conditions, the BZND model, represented by Equations (2) and  (4), can be reformulated as

$$\begin{array}{cc} \; & A{\left(t\right)}^{T}\dot{Z}\left(t\right)+\dot{Z}\left(t\right)A\left(t\right)=-\dot{A}{\left(t\right)}^{T}Z\left(t\right)-Z\left(t\right)\dot{A}\left(t\right)-\dot{B}\left(t\right)\\ & -\gamma F_{1}\left(A{\left(t\right)}^{T}Z\left(t\right)+Z\left(t\right)A\left(t\right)+B\left(t\right)\right)\\ & -\lambda_{1}F_{2}\left(A{\left(t\right)}^{T}Z\left(t\right)+Z\left(t\right)A\left(t\right)+B\left(t\right)\right)\\ & -\gamma \int_{0}^{t}F_{1}\left(A{\left(\iota \right)}^{T}Z\left(\iota \right)+Z\left(\iota \right)A\left(\iota \right)+B\left(\iota \right)\right)d\iota )+v\left(t\right). \end{array}$$

Lei constructed a model based on the IEZNN design formula to address the TVSE problem [95], and the error monitoring function is

E(t)=L(t)Z(t)−Z(t)F(t)+G(t).

Here, L(t), F(t), and G(t) are given matrices, while Z(t) is the unknown time-varying matrix to be determined. The design process for the noise-resistant integrated enhanced zeroing neural network (NIEZNN) model is outlined below.

The design formula, as presented in Equation (4), is employed to solve the TVSE. To further enhance the model’s anti-interference capability, the NIEZNN model is extended to incorporate additional random noise, resulting in the noise-augmented NIEZNN model. The extended model is expressed as follows:

(5) $$\begin{array}{cc} \; & L\left(t\right)\dot{Z}\left(t\right)-\dot{Z}\left(t\right)F\left(t\right)=Z\left(t\right)\dot{F}\left(t\right)-\dot{L}\left(t\right)Z\left(t\right)-\dot{G}\left(t\right)\\ & -\gamma F_{1}\left(L\left(t\right)Z\left(t\right)-Z\left(t\right)F\left(t\right)+G\left(t\right)\right)\\ & -\lambda_{1}F_{2}\left(L\left(t\right)Z\left(t\right)-Z\left(t\right)F\left(t\right)+G\left(t\right)\right)\\ & -\gamma \int_{0}^{t}F_{1}\left(L\left(\iota \right)Z\left(\iota \right)-Z\left(\iota \right)F\left(\iota \right)+G\left(\iota \right)\right)d\iota )+v\left(t\right). \end{array}$$

This model exhibits exceptional performance in solving TVSE, particularly demonstrating significant robustness and noise resilience across a range of noisy environments. Furthermore, when applied to time-varying problems, especially in TVQO [96,97], and other time-varying issues under noisy conditions [93], the IEZNN outperforms the traditional ZNN model, offering superior robustness, noise resistance, and computational accuracy.

### 2.3. Design of the Double Integral-Enhanced Zeroing Neural Network Model

In scientific computing, tasks such as TVMI, solving linear equations, and other similar problems frequently encounter noise interference, including constant noise, linear noise, and random noise. While traditional ZNN models and the IEZNN can suppress certain types of noise, they remain susceptible to computational inaccuracies when faced with linear or more complex noise forms. To address this limitation, it is essential to incorporate a double integral feedback mechanism, which further mitigates long-term bias and enhances the performance of the model.

The double integral term improves the system’s ability to detect and address error accumulation. This feedback mechanism accelerates error correction, thereby enhancing the network’s convergence rate. Through double integral control, the system’s error function becomes sensitive not only to the current error (instantaneous feedback) and accumulated historical errors (single integral feedback), but also adjusts for more complex error accumulation patterns (double integral feedback). This structure is further elaborated in the article [98]. The multi-level feedback mechanism strengthens the network’s dynamic stability, enhancing its robustness in complex environments.

The design formula for the double integral enhanced zeroing neural network (DIEZNN) is given as follows:

(6) $$\begin{array}{c} \dot{E}\left(t\right)=-\gamma E\left(t\right)-\lambda_{1}\int_{0}^{t}E\left(\iota \right)d\iota -\\ \lambda_{2}\int_{0}^{t}\int_{0}^{\iota }E\left(\eta \right)d\eta d\iota , \end{array}$$

where γ>0 and $\lambda_{1}>0$, $\lambda_{2}>0$ are design parameters. The first integral term $\int_{0}^{t}E\left(\iota \right)\;d\iota$ compensates for global error accumulation. The second integral term $\int_{0}^{t}\int_{0}^{\iota }E\left(\eta \right)\;d\eta \;d\iota$ addresses the deeper correction of the error’s changing trend.

The introduction of double integrals effectively addresses such problems. Liao constructed a DIEZNN model based on a novel integral design formula, which inherently possesses linear noise tolerance [99]. To monitor the TVMI problem, the error function is designed in the same manner as in Equation (1), In this context, x(t) represents the system state that needs to be solved. Although the IIEZNN model can suppress noise to some extent, it still exhibits limitations when handling linear noise. Therefore, a new model is required to address the presence of linear noise. Liao *et al*. derived the design formula for the DIEZNN model as follows:

(7) $$\begin{array}{c} \dot{E}\left(t\right)=-\gamma E\left(t\right)-\lambda_{1}\int_{0}^{t}E\left(\iota \right)d\iota -\\ \lambda_{2}\int_{0}^{t}\int_{0}^{\iota }E\left(\eta \right)d\eta d\iota +v\left(t\right). \end{array}$$

Further, the DIEZNN model is as follows:

$$\begin{array}{cc} \; & B\left(t\right)\dot{Y}\left(t\right)=-\dot{B}\left(t\right)Y\left(t\right)-\gamma \left(B\left(t\right)Y\left(t\right)-I\right)\\ & -\lambda_{1}\int_{0}^{t}\left(B\left(t\right)Y\left(t\right)-I\right)d\iota \\ & -\lambda_{2}\int_{0}^{t}\int_{0}^{\iota }\left(B\left(\eta \right)Y\left(\eta \right)-I\right)d\eta d\iota +v\left(t\right). \end{array}$$

The article conducted two simulation case studies with varying matrix dimensions and linear noise. Both the theoretical proof and the simulation examples thoroughly demonstrate the inherent linear noise suppression capability of the DIEZNN model.

The double-integral structure possesses a stronger cumulative filtering capability, effectively attenuating both high- and low-frequency noise compared to the single-integral model. In control theory, the integration operation inherently exhibits low-pass filtering characteristics. First-order integration can suppress high-frequency disturbances but is limited in mitigating slowly varying noise. By introducing second-order integration, the system gains enhanced temporal smoothing ability, enabling more accurate extraction of the true error.

**Figure 2:**
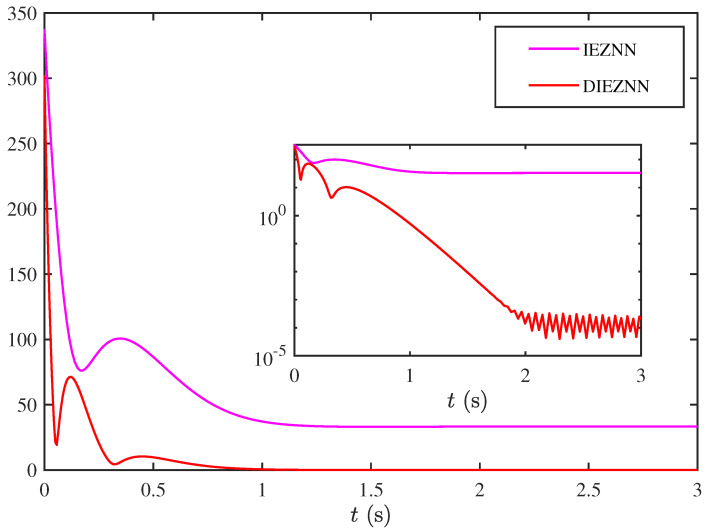
Real-time error plots of IEZNN and DIEZNN under linear noise conditions.

This structure delays the impact of instantaneous noise, suppresses error propagation, and significantly enhances the robustness and stability of the system. To validate the design motivation, this paper includes a comparative example of the IEZNN and DIEZNN under linear noise conditions. Figure 2 clearly demonstrates the superiority of the DIEZNN in terms of error convergence and noise resistance.

The DIEZNN model, by introducing a proportional-integral-double integral control mechanism, demonstrates significant advantages in solving dynamic computational problems such as TVMI [100], time-varying linear equations, MOO, embedded real-time computation, and control. With its exceptional noise resistance, rapid convergence, and adaptability to various environments, the DIEZNN offers an efficient and reliable solution for dynamic system modeling, control, and optimization. It has contributed to technological advancements and broadened the scope of applications in the field of dynamic computation.

Therefore, the improvements in the ZNN model structure can be summarized as follows: Through the iterative evolution from the traditional ZNN to the enhanced IEZNN, and then to the DIEZNN, these advancements have significantly improved the model’s robustness to noise and its interference resistance. This progression has enabled the model to be effectively applied in complex multi-noise scenarios and laid the foundation for the further development of subsequent models.

This paper discusses the single-integral and double-integral ZNN models. The single-integral model improves both stability and convergence. The double-integral model, by incorporating a dual-feedback mechanism, enhances noise resistance and accelerates convergence. Although the t-fold integral model could potentially further improve robustness or trajectory smoothness, its increased complexity introduces a higher computational burden, which may lead to response delays and numerical stability issues in real-time systems. Consequently, this model is not considered in this paper.

## 3. Activation Functions of Zeroing Neural Network Model and Other Enhancements

Although optimizations based on model architectures have significantly enhanced the robustness of neural networks in noisy environments, the improvement in model convergence speed still faces certain limitations. Consequently, researchers have shifted their focus to optimizing activation functions, with the goal of further enhancing the model’s convergence performance and computational efficiency through the design and introduction of more effective activation functions.

### 3.1. Nonlinear Activation Functions with Enhanced Convergence Properties

From the perspective of convergence speed, three common types of convergence can be distinguished: finite-time convergence, fixed-time convergence, and predefined-time convergence. These types all involve the rate at which system errors converge, but they differ in their specific characteristics. Finite-time convergence refers to a dynamic system’s ability to reach its target state or ideal solution within a finite time, typically with the target solution being zero or sufficiently close to zero. Fixed-time convergence refers to a system behavior that ensures the system state converges to the equilibrium point within a finite time, with the convergence time being independent of the initial conditions. However, the actual convergence time is only guaranteed to have an upper bound, and it cannot be explicitly predetermined. In contrast, preset-time convergence describes a framework in which the convergence time is determined during the design phase. Unlike fixed-time convergence, preset-time convergence ensures that the system converges within a user-specified time frame, with the convergence time being adjustable to meet design requirements, thus offering stronger guarantees in time control than fixed-time convergence.

A large number of activation functions have been proposed to accelerate convergence. Finite-time convergence is primarily grounded in Lyapunov stability theory. By constructing an appropriate Lyapunov function, it has been demonstrated that the error or objective function can converge to zero within a finite time. Nonlinear activation functions play a pivotal role in the design of neural networks that achieve finite-time convergence and are extensively utilized in numerous neural network models endowed with finite-time convergence properties [51]. These activation functions significantly improve convergence by altering both the rate and direction of error reduction.

In the literature [101], Xiao constructed a finite-time convergence model, with the error function defined as

(8) E(t)=Y(t)N(t)−I.

The expression of the model is as follows:

(9) $$\dot{E}\left(t\right)=-\gamma F\left(E\left(t\right)\right).$$

Considering Equations (8) and  (9), the ZNN-A model is as follows:

$$\begin{array}{c} \dot{Y}\left(t\right)N\left(t\right)=-Y\left(t\right)\dot{N}\left(t\right)-\gamma F\left(Y\left(t\right)N\left(t\right)-I\right). \end{array}$$

The sign-bi-power (SBP) activation function is defined as follows:

$$\begin{array}{c} F(y_{ij})=\frac{1}{2}\left({\mathrm{Lip}}^{a}\left(y_{ij}\right)+{\mathrm{Lip}}^{1/a}\left(y_{ij}\right)\right), \end{array}$$

$$\begin{array}{c} {\mathrm{Lip}}^{a}\left(y_{ij}\right)=\left\{\begin{array}{cc} |y_{ij}{|}^{a}, & \mathrm{if}\;y_{ij}>0,\\ 0, & \mathrm{if}\;y_{ij}=0,\\ -|y_{ij}{|}^{a}, & \mathrm{if}\;y_{ij}<0. \end{array}\right. \end{array}$$

The upper bound of the convergence time for this model is

$$\begin{array}{c} \max\left\{\frac{2|e^{-}{\left(0\right)|}^{1-a}}{\varrho (1-a)},\frac{2|e^{+}{\left(0\right)|}^{1-a}}{\varrho (1-a)}\right\}. \end{array}$$

In the formula, e(0) denotes the initial error.

Liao proposed a novel complex-valued zeroing neural network (NCZNN) [53,102], which achieves finite-time convergence in the complex domain through two distinct approaches. The error function is

(10) E(t)=B(t)Y(t)−k(t).

Considering Equation (9), the NCZNN model is given by

$$\begin{array}{c} B\left(t\right)\dot{Y}\left(t\right)=-B\left(t\right)\dot{Y}\left(t\right)-\gamma F\left(B\left(t\right)Y\left(t\right)-k\left(t\right)\right)+\dot{k}\left(t\right). \end{array}$$

In general, there are two approaches for handling complex-valued activation functions, as follows:

$$\begin{array}{c} F_{1}\left(C+iH\right)=\Lambda \left(C\right)+i\Lambda \left(H\right). \end{array}$$

$$\begin{array}{c} F_{2}\left(C+iH\right)=\Lambda \left(\tau \right)\circ \exp\left(i\Delta \right). \end{array}$$

The upper bound of the NCZNN model is given as follows:

$$\begin{array}{c} t_{C}\leq \frac{{\left|a\left(0\right)\right|}^{1-m}}{\gamma (1-m)}, \end{array}$$

where, $a\left(0\right)=\max_{k}\left\{|e_{k}\left(0\right)|\right\}$. In comparative experiments, the NCZNN model consistently outperforms the CZNN model [102].

In reference [95], Lei et al. introduced a nonlinear activation integral-enhanced zeroing neural network (NIEZNN) model based on the coalescent activation function (C-AF) activation function, comparing it with existing ZNN models. The experimental results highlighted its superiority.

In reference [103], Xiao et al. investigated the time-varying inequality constrained quaternion matrix least squares (TVIQLS) problem and proposed a fixed-time noise-tolerant zeroing neural network (FTNTZNN) model to solve it in complex environments. The TVIQLS problem is reformulated into matrix form, that is, the error function is analogous to Equation (10). By combining the error equation and the design Equation (4), the FTNTZNN model is formulated as follows:

$$\begin{array}{c} \dot{E}\left(t\right)=-\gamma F\left(E\left(t\right)\right)-\lambda_{1}\left(\gamma \int_{0}^{t}F\left(E\left(\iota \right)\right)\;d\iota +F\left(E(t)\right)\right). \end{array}$$

When solving the TVIQLS problem, only finite-time convergence can be achieved, and not fixed-time convergence. To address both challenges simultaneously, an improved activation function F(·) is integrated into the ZNN model, defined as follows:

$$\begin{array}{c} F_{l}\left(z\right)=\left\{\begin{array}{cc} \xi_{1}{\left|z\right|}^{\mu }\mathrm{sign}\left(z_{l}\right)+\xi_{2}z_{l}, & \mathrm{if}\;|z_{l}|\leq \;1\\ \xi_{2}{\left|z\right|}^{\mu }\mathrm{sign}\left(z_{l}\right)+\xi_{3}z_{l}. & \mathrm{if}\;|z_{l}|>1 \end{array}\right. \end{array}$$

Here, $0<\mu_{1}<1$ and $\mu_{2}>1$, $\xi_{1},\xi_{2},\xi_{3}$ are positive parameters, and the upper bound of the model’s convergence is given by

$$\begin{array}{c} T=T_{1}+T_{2}\leq \frac{\rho +\lambda }{\rho \lambda \xi_{1}\left(1-\mu_{1}\right)}+\frac{\rho +\lambda }{\rho \lambda \xi_{2}\left(\mu_{2}-1\right)}. \end{array}$$

The FTNTZNN model is robust to initial values and external noise, offering a significant advantage over traditional zeroing neural network (CZNN) models. When compared to other ZNN models employing conventional activation functions, the FTNTZNN model exhibits faster convergence and enhanced robustness.

Xia et al. incorporated the activation function [36] into the ZNN model, achieving fixed-time convergence. Its form is as follows:

$$\begin{array}{c} F\left(x\right)=\frac{1}{2}h_{1}{\mathrm{wsbp}}^{b}\left(x\right)+\frac{1}{2}h_{2}{\mathrm{wsbp}}^{1/b}\left(x\right)+\frac{1}{2}h_{3}x. \end{array}$$

where $h_{1},h_{2},h_{3}>0$, b∈(0,1), and the function wsbp(·) is defined as

$$\begin{array}{c} {\mathrm{wsbp}}^{b}\left(y\right)=\left\{\begin{array}{cc} {\left|y\right|}^{b}, & \mathrm{if}\;y>0\\ 0, & \mathrm{if}\;y=0\\ -{\left|y\right|}^{b}. & \mathrm{if}\;y<0 \end{array}\right. \end{array}$$

Define the error function as

$$\begin{array}{c} E(t)=A(t)A(t)Z(t)-L(t)Y(t). \end{array}$$

The design formula is identical to that in Equation (9), i.e., the corresponding first-order fixed-time ZNN model (FOZNN-1) is

$$\begin{array}{c} A\left(t\right)A\left(t\right)\dot{Z}\left(t\right)=\dot{A}\left(t\right)Y\left(t\right)+A\left(t\right)\dot{Y}\left(t\right)\\ -\dot{A}\left(t\right)A\left(t\right)Z\left(t\right)-A\left(t\right)\dot{A}\left(t\right)Z\left(t\right)\\ -\gamma F(A(t)A(t)Z(t)-A(t)Y(t)). \end{array}$$

It can be concluded that the upper bound of its convergence time is

$$\begin{array}{c} T_{1}\leq \left\{\begin{array}{cc} \frac{\mu }{n_{a}\left(b-g\right)}\log\left(\frac{\left|b\right|}{\left|g\right|}\right), & n_{c}>2\sqrt{n_{a}n_{b}}\\ \frac{\mu }{\sqrt{n_{a}n_{b}}}\frac{k}{1-k}, & n_{c}=2\sqrt{n_{a}n_{b}}\\ \frac{\mu }{n_{a}k_{1}}\left(\frac{\pi }{2}-\tan^{-1}k_{2}\right), & 0<n_{c}<2\sqrt{n_{a}n_{b}}\\ \frac{\mu \pi }{2\sqrt{n_{a}n_{b}}}, & n_{c}\leq 0 \end{array}\right. \end{array}$$

where $n_{a}$, $n_{b}$, $n_{c}$, *b*, and *g* are parameters, and 0<k<1. The values −b and −g are the solutions of

$$r\left(s\right)=n_{a}s^{2}-n_{c}s+n_{b},$$

with

$$k_{1}=\frac{\sqrt{4n_{a}n_{b}-n_{c}^{2}}}{4n_{a}^{2}},\;k_{2}=\frac{n_{c}}{\sqrt{4n_{a}n_{b}-n_{c}^{2}}}.$$

The experiment shows that, compared to other models, this model achieves stronger convergence performance and realizes fixed-time convergence.

In the literature [86], Xiao introduced a versatile activation function (VAF) to address the TVMI problem. Considering Equations (1) and (9), the model can be expressed as follows:

(11) $$\begin{array}{c} B\left(t\right)\dot{Y}\left(t\right)=-B\left(t\right)\dot{Y}\left(t\right)-\gamma F\left(B\left(t\right)Y\left(t\right)-I\right)+S\left(t\right), \end{array}$$

where S(t) represents general noise, and the design formula for the activation function is as follows:

$$\begin{array}{c} F\left(x\right)=(r_{1}{\left|x\right|}^{\epsilon }+r_{2}{\left|x\right|}^{\zeta })\mathrm{sgn}\left(x\right)+r_{3}x+r_{4}\mathrm{sgn}\left(x\right). \end{array}$$

The upper bound is given by

$$\begin{array}{c} t_{r}=\frac{1}{\varsigma (1-\epsilon )}+\frac{1}{\upsilon (\zeta -1)}, \end{array}$$

where ς>0 and υ>0.

In the literature [104], Li et al. were the first to achieve predefined-time convergence for the ZNN model by introducing two novel activation functions. The error function is defined as follows:

$$\begin{array}{c} E\left(t\right):=Y^{2}\left(t\right)-N\left(t\right). \end{array}$$

Given the dynamic matrix N(t) and the system dynamics Y(t) to be solved, the perturbation time-varying ZNN (PTZNN) model is expressed as follows:

$$\begin{array}{c} Y\left(t\right)\dot{Y}\left(t\right)+\dot{Y}\left(t\right)Y\left(t\right)=-\lambda F(Y^{2}\left(t\right)\\ -N\left(t\right))-\dot{N}\left(t\right)+G\left(t\right). \end{array}$$

To achieve predefined-time convergence, two activation functions are proposed, which are defined as follows:

$$\begin{array}{c} F_{1}\left(x\right)=(\kappa_{1}{\left|x\right|}^{p}+\kappa_{2}{\left|x\right|}^{q})\mathrm{sign}\left(x\right)+\kappa_{3}x+\kappa_{4}\mathrm{sign}\left(x\right). \end{array}$$

The design formula for the second activation function is as follows:

$$\begin{array}{c} F_{2}\left(x\right)=\gamma_{1}\exp\left({\left|x\right|}^{p}\right){\left|x\right|}^{1-p}\mathrm{sign}\left(x\right)/p+\gamma_{2}x+\gamma_{3}\mathrm{sign}\left(x\right). \end{array}$$

The upper bound of the convergence time derived from the first activation function is as follows:

$$\begin{array}{c} t_{r}\leq \frac{1}{\lambda \kappa_{1}\left(1-p\right)}+\frac{1}{\lambda \kappa_{2}\left(q-1\right)}. \end{array}$$

When utilizing the second activation function, the upper bound of the convergence time is

$$\begin{array}{c} t_{c}\leq \frac{1}{\lambda \gamma_{1}}. \end{array}$$

In addressing the dynamic matrix square root (DMSR) problem, the PTZNN model outperforms existing models in both convergence and robustness.

In reference [105], Li et al. proposed a strict predefined-time convergence and noise-tolerant ZNN (SPTC-NT-ZNN) for solving time-varying linear systems. The model is consistent with Equation (11), where the activation function is defined as

$$\begin{array}{c} h\left(\delta \right):=\left\{\begin{array}{cc} \delta /(t_{c}-t), & t\in [0,t_{c})\\ \delta +{\left|\delta \right|}^{p}\mathrm{sign}\left(\delta \right)+\xi \mathrm{sign}\left(\delta \right), & t\in [t_{c},+\infty ) \end{array}\right. \end{array}$$

where the parameters 0<p<1 and ξ≥0 are given, and the parameter $t_{c}$ is related to the convergence time. Additionally, the function $\mathrm{sign}\left(\delta \right)=\frac{\delta }{\left|\delta \right|}$ is defined for δ≠0.

This ensures the required timely convergence and robustness for time-critical applications. The strict predefined-time convergence and noise tolerance of the SPTC-NT-ZNN have been theoretically proven and further validated through comparative experiments to demonstrate its superiority. The comparison focused on two illustrative problems: TVOLE and TVULE. The numerical results demonstrate that, in both convergence and robustness, the SPTC-NT-ZNN outperforms other existing ZNN models in solving these problems.

**Remark** **1.** *In ZNN, nonlinear activation functions play a crucial role in shaping the neural dynamics, facilitating the achievement of the desired convergence behavior. These functions typically operate on error terms and, through careful design, can guide the system to systematically reduce the error to zero. The convergence behaviors influenced by well-designed nonlinear activation functions include finite-time convergence, fixed-time convergence, and preset-time convergence.*

**Remark** **2.** *This section presents the mathematical definitions and fundamental properties of three distinct types of convergence: finite-time convergence, fixed-time convergence, and predefined-time convergence.*

Finite-time convergence refers to the system’s convergence to the equilibrium point x=0 from an initial state $x_{0}$ within a finite time. If the system is Lyapunov stable, and there exists a finite convergence time function $T(x_{0})$ depending on the initial state $x_{0}$, then the system will converge within that time. Specifically, given the system’s dynamics,

$$\dot{x}\left(t\right)=f\left(x\left(t\right),t\right),\;x\left(0\right)=x_{0},$$

the finite-time stability satisfies the following condition:

$$\dot{V}\left(x\right)\leq -kV{\left(x\right)}^{q},\;0<q<1$$

where V(x) is a positive definite Lyapunov function, and *k* is a constant. The convergence time is

$$T\left(x_{0}\right)=\frac{1}{k(1-q)}V{\left(x_{0}\right)}^{1-q}$$

This time explicitly depends on the initial condition $x_{0}$ and indicates that the system will converge to the equilibrium point within a finite time.

Fixed-time convergence refers to the system’s convergence to the equilibrium point in a fixed, initial-condition-independent time. For all initial conditions $x_{0}$, there exists a fixed maximum convergence time $T_{\max}$ such that

$$x\left(t\right)=0,\;\forall t\geq T_{\max}$$

A typical Lyapunov condition is

$$\dot{V}\left(x\right)\leq -{\left(\alpha V{\left(x\right)}^{p}+\beta V{\left(x\right)}^{q}\right)}^{k}$$

where *p*, *q* are constants satisfying pk<1 and qk>1, and constants α,β>0. The upper bound for the fixed-time convergence is

$$T_{\max}=\frac{1}{\alpha^{k}\left(1-pk\right)}+\frac{1}{\beta^{k}\left(qk-1\right)}$$

This upper bound is independent of the initial condition $x_{0}$ and ensures that the system converges in fixed time.

Predefined-time convergence requires that the system fully converges to the equilibrium point within a user-specified fixed time $T_{p}$, regardless of the initial condition. The system’s Lyapunov condition is

$$\dot{V}\left(x\right)\leq -\frac{1}{pT_{p}}e^{V{\left(x\right)}^{p}}V{\left(x\right)}^{1-p}$$

where *p* is a constant, satisfying 0<p≤1. If this condition holds, the system exhibits strong predefined-time stability, and the convergence time is strictly $T_{p}$.

**Figure 3:**
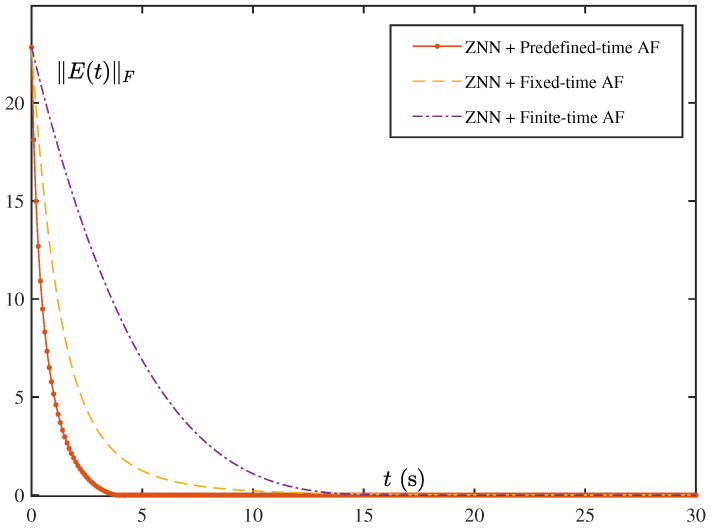
Real-time errors of the three different models.

The Figure 3 illustrates the convergence behavior of the ZNN error ${∥E\left(t\right)∥}_{F}$ over time under various activation functions. Among them, the orange curve—corresponding to the predefined-time activation function—achieves the fastest convergence, followed by the yellow curve representing the fixed-time activation function. In contrast, the purple curve, associated with the finite-time activation function, exhibits the slowest convergence. These results clearly indicate that the predefined-time activation function facilitates the most rapid error reduction in the ZNN, outperforming the fixed-time and finite-time counterparts. This observation underscores the significant influence of activation function design on the convergence performance of the ZNN.

### 3.2. Nonlinear Activation Functions with Noise-Tolerant Capabilities

With ongoing advancements in neural network models, nonlinear activation functions have become crucial not only for accelerating convergence but also for significantly enhancing noise robustness. Specifically, noise robustness refers to the model’s ability to maintain stability and perform inference and prediction effectively in the presence of input noise or system disturbances.

In summary, nonlinear activation functions enhance the model’s noise robustness, enabling the network to effectively handle complex and uncertain environments in real-world applications. Simultaneously, they accelerate convergence while preserving the model’s efficiency and robustness. This combination lays a strong foundation for the widespread adoption of neural networks in fields such as real-time control, intelligent decision making, and dynamic optimization.

In reference [106], Xiao et al. constructed and analyzed a novel recursive neural network (NRNN) that exhibits finite-time convergence and exceptional robustness, specifically for solving the TVSE with additive noise. In contrast to the design methodology of the ZNN, the proposed NRNN utilizes a sophisticated integral design formula in conjunction with a nonlinear activation function. This integration not only accelerates the convergence rate but also effectively mitigates the impact of unknown additive noise in the process of solving dynamic Sylvester equations. The integral design formula is analogous to Equation (4). The design of the activation function is as follows:

$$\begin{array}{c} F_{1}\left(e_{ij}\right)=F_{2}\left(e_{ij}\right)=\varphi^{u}\left(e_{ij}\right)+\varphi^{1/v}\left(e_{ij}\right), \end{array}$$

where the design parameters 0<v<1, The definition of $\varphi^{v}\left(\cdot \right)$ is given by

$$\begin{array}{c} \varphi^{v}\left(e_{ij}\right)=\left\{\begin{array}{cc} |e_{ij}{|}^{v}, & \mathrm{if}\;e_{ij}>0\\ 0, & \mathrm{if}\;e_{ij}=0\\ -|e_{ij}{|}^{v}, & \mathrm{if}\;e_{ij}<0 \end{array}\right. \end{array}$$

The ij-th subsystem of the integral design formula can be expressed as follows:

$$\begin{array}{c} {\dot{e}}_{i}\left(t\right)=-\gamma f_{1}\left(e_{ij}\left(t\right)\right)-\lambda f_{2}\left(\left(e_{ij}\left(t\right)\right)+\gamma \int_{0}^{t}f_{1}\left(e_{ij}\left(\iota \right)\right)\;d\iota \right). \end{array}$$

By combining the error function with the design formula, the NRNN model can be constructed to solve the dynamic Sylvester equation, which has a similar form to Equation (5).

In [81], Xiao et al. applied the ZNN model to solve TVSME. The use of a noise-resistant activation function allows the ZNN model to effectively solve the Stein equation in noisy environments. Therefore, the ZNN model not only exhibits enhanced convergence performance but also improves noise immunity. To address this issue, a complex-valued error function is defined:

$$\begin{array}{c} E(t)=D(t)Y(t)Z(t)+Y(t)-F(t), \end{array}$$

By utilizing the Kronecker product, the error function E(t) can be reformulated as

$$\begin{array}{c} \vec{E}\left(t\right)=V\left(t\right)\vec{Y}\left(t\right)-\vec{F}\left(t\right). \end{array}$$

Since a complex number can be expressed as the sum of its real and imaginary parts, $\vec{E}\left(t\right)$ is represented as ${\vec{E}}_{r}\left(t\right)+i{\vec{E}}_{i}\left(t\right)$, where *i* is the imaginary unit. Furthermore, we have

$$\begin{array}{c} \vec{E}\left(t\right)=-\varrho \left(t\right)\left(F\left({\vec{E}}_{r}\left(t\right)\right)+iF\left({\vec{E}}_{i}\left(t\right)\right)\right). \end{array}$$

To ensure noise robustness, the following activation function is adopted:

$$\begin{array}{c} F\left(x\right)=\frac{r_{1}}{d}{\exp(|x|}^{d}{)|x|}^{1-d}\mathrm{sign}\left(x\right). \end{array}$$

The PTAN-VP ZNN model presented below can be derived using the design formula outlined above, as detailed in [107].

$$\begin{array}{c} \left\{\begin{array}{c} V\left(t\right)\dot{\vec{Y}}\left(t\right)=-\dot{V}\left(t\right)\vec{Y}\left(t\right)+\dot{\vec{F}}\left(t\right)\\ \;\;-\varrho \left(t\right)(F\left({\vec{E}}_{r}\left(t\right)\right)+iF\left({\vec{E}}_{i}\left(t\right)\right)),\\ \dot{\gamma }\left(t\right)=\exp(\alpha \mathrm{sign}(|\vec{E}\left(t\right)|))-1. \end{array}\right. \end{array}$$

Compared to other ZNN models, such as the LZNN [108], NLZNN [109], FTCZNN [72] and PTCZNN [104], the PTAN-VP ZNN exhibits superior interference rejection performance. This paper presents a theoretical analysis of the stability and robustness of the PTAN-VP ZNN. The validity of the theoretical results has been confirmed through numerical simulations, which also highlight the advantages of the PTAN-VP ZNN. Moreover, the PTAN-VP ZNN has been successfully applied to mobile robotic arms, demonstrating its potential for use in robotic control.

In [97], a nonlinear activation-based integral design formula was proposed to address the effects of additive noise. Building upon this design formula, a NRNN was developed to solve dynamic quadratic optimization problems. Compared to the ZNN applied to this problem, the proposed RNN model demonstrates significant finite-time convergence and inherent noise-resistance capabilities.

The activation functions and their convergence types are shown in Table 1. In addition to using activation functions to improve the robustness of the model, adaptive compensation terms can be introduced to mitigate the impact of noise. For example, Liao et al. [47] proposed a harmonic noise-tolerant zeroing neural network (HNTZNN) model to efficiently solve matrix pseudoinversion problems.

Algorithm 1 presents the pseudocode in a unified format, illustrating the discrete-time implementation process of four ZNN models: the original ZNN model, the ZNN model enhanced with nonlinear activation functions, the IEZNN model, and the DIEZNN model. This algorithmic framework is suitable for typical application scenarios such as real-time control systems, trajectory tracking, robotic control, and the solution of dynamic matrix equations.

The pseudocode mainly consists of the following components:

- **Parameter initialization**: including the initial state Y(0);
- **Time-step iteration**: iterating from n=0 to $t_{\max}/\tau$ with a fixed step size τ;
- **Model-specific control law and state update**: updating the state variable $Y(t_{n})$ based on the corresponding control law of each ZNN model;
- **Introduction and update of auxiliary variables**: where $Z(t_{n})$ denotes the single-integral term and $N(t_{n})$ denotes the double-integral term.

### 3.3. The Variable Parameter Improves the Convergence Performance of Zeroing Neural Network Models

To enhance the convergence rate, the use of variable parameters (VPs) presents another effective strategy. These parameters are dynamically adjusted over time, typically following a time-dependent function (e.g., exponential or power functions) that governs their evolution. The dynamic adjustment capability of VPs offers significant benefits, including improved system convergence, enhanced robustness, and better alignment with practical hardware constraints. Although the design and implementation may be more complex, these advantages make variable parameters the preferred approach for addressing complex dynamic problems, particularly in scenarios characterized by time-varying properties or external disturbances.

In the literature [52], a variable-parameter recurrent neural network (VP-CDNN) is proposed, and Equation (9) is reformulated as follows:

$$\begin{array}{c} \dot{E}\left(t\right)=-\chi \left(t\right)F\left(E\left(t\right)\right)=-\left(t^{\kappa }+\kappa \right)F\left(E\left(t\right)\right). \end{array}$$

**Algorithm 1:** Pseudocode of Discrete Controllers Based on Different ZNN Models**Parameters initialization:** e.g., Y(0)
**for** n=0 to $t_{\max}/\tau$ **do**      Compute relevant coefficients with $t_{n}=n\tau$, e.g., $Q\left(t_{n}\right),\gamma ,\lambda_{1},\lambda_{2}$.      **if** (ZNN controller I) **then**            Update $Y(t_{n+1})$ by following the control law:$$\dot{Y}\left(t_{n}\right)=B^{†}\left(-\gamma \left(BY\left(t_{n}\right)-K\left(t_{n}\right)\right)+\dot{K}\left(t_{n}\right)\right)$$$$Y\left(t_{n+1}\right)=Y\left(t_{n}\right)+\tau Y\left(t_{n}\right)$$      **else if** (ZNN controller II) **then**            Update $Y(t_{n+1})$ by following the control law:$$\dot{Y}\left(t_{n}\right)=B^{†}\left(-\gamma F\left(BY\left(t_{n}\right)-K\left(t_{n}\right)\right)+\dot{K}\left(t_{n}\right)\right)$$$$Y\left(t_{n+1}\right)=Y\left(t_{n}\right)+\tau Y\left(t_{n}\right)$$      **else if** (IEZNN controller under noise-free conditions) **then**          Update $Y(t_{n+1})$ by following the control law:$$\dot{Y}\left(t_{n}\right)=B^{†}\left(-\gamma F\left(BY\left(t_{n}\right)-K\left(t_{n}\right)\right)+Z\left(t_{n}\right)\right)+\dot{K}\left(t_{n}\right)$$$$\dot{Z}\left(t_{n}\right)=-\lambda_{1}F\left(BY\left(t_{n}\right)-K\left(t_{n}\right)\right)$$$$Z\left(t_{n+1}\right)=Z\left(t_{n}\right)+\tau \dot{Z}\left(t_{n}\right)$$$$Y\left(t_{n+1}\right)=Y\left(t_{n}\right)+\tau \dot{Y}\left(t_{n}\right)$$      **else if** (DIEZNN controller under noisy conditions) **then**            Update $Y(t_{n+1})$ by following the control law:$$\dot{Y}\left(t_{n}\right)=B^{†}\left(-\gamma F\left(BY\left(t_{n}\right)-K\left(t_{n}\right)\right)+Z\left(t_{n}\right)+N\left(t_{n}\right)\right)+\dot{K}\left(t_{n}\right)$$$$\dot{N}\left(t_{n}\right)=\lambda_{2}/\lambda_{1}*Z\left(t_{n}\right)$$$$\dot{Z}\left(t_{n}\right)=-\lambda_{1}F\left(BY\left(t_{n}\right)-K\left(t_{n}\right)\right)$$$$N\left(t_{n+1}\right)=N\left(t_{n}\right)+\tau \dot{N}\left(t_{n}\right)$$$$Z\left(t_{n+1}\right)=Z\left(t_{n}\right)+\tau \dot{Z}\left(t_{n}\right)$$$$Y\left(t_{n+1}\right)=Y\left(t_{n}\right)+\tau \dot{Y}\left(t_{n}\right)$$      **end if****end for**


Further, the CDNN model is obtained as follows:

$$\begin{array}{cc} \; & L\left(t\right)\dot{Z}\left(t\right)-\dot{Z}\left(t\right)F\left(t\right)=Z\left(t\right)\dot{F}\left(t\right)-\dot{L}\left(t\right)Z\left(t\right)-\dot{G}\left(t\right)\\ & -\left(t^{\kappa }+\kappa \right)F\left(L\left(t\right)Z\left(t\right)-Z\left(t\right)F\left(t\right)+G\left(t\right)\right). \end{array}$$

This paper presents a novel VP-CDNN model, which innovatively incorporates a time-varying parameter function χ(t), significantly enhancing the solving performance of time-varying Sylvester equations. Through rigorous mathematical proofs, the study confirms the superior performance of this model in terms of convergence and robustness. Specifically, the VP-CDNN not only achieves super-exponential convergence performance but also demonstrates strong robustness characteristics, all of which have been thoroughly validated through multiple theorems. In the comparative simulation experiments, the VP-CDNN exhibited significant advantages in convergence speed.

In the literature [56], Xiao constructed an innovative finite-time varying-parameter convergent differential neural network (FT-VP-CDNN) aimed at solving nonlinear and non-convex optimization problems. The study not only provides a detailed analysis of the network’s performance but also presents its design formula, which is specifically expressed as follows:

$$\begin{array}{c} \dot{E}\left(t\right)=-\vartheta \left(t\right)F\left(E\left(t\right)-E^{+}\left(t\right)+\tilde{E}\left(t\right)\right), \end{array}$$

where $\vartheta \left(t\right)=\varepsilon \exp\left(t\right)=\varepsilon e^{t}>0$ represents a time-varying parameter function. Research indicates that the proposed finite-time varying-parameter convergent differential neural network (FT-VP-CDNN) demonstrates significant performance advantages over the finite-time fixed-parameter convergent differential neural network (FT-FP-CDNN).

In [120], an IEZNN model was proposed to address the TVMI under noise interference. The IEZNN model performs well in handling relatively small time-varying noise; however, its performance is significantly affected by noise interference. As the noise level increases, the convergence accuracy of the model may degrade, and it may even fail to accurately approximate the theoretical solution. To address this limitation and further enhance performance, Xiao constructed a novel variable-parameter noise-tolerant zeroing neural network (VPNTZNN) model in this study. The mathematical formulation of the model is presented as follows:

$$\begin{array}{cc} \; & B\left(t\right)\dot{Y}\left(t\right)=-\dot{B}\left(t\right)Y\left(t\right)-\mu_{1}\left(t\right)\left(B\left(t\right)Y\left(t\right)-I\right)\\ & -\mu_{2}\left(t\right)\int_{0}^{t}\left(B\left(t\right)Y\left(t\right)-I\right)d\tau +D\left(t\right). \end{array}$$

The design formula is derived from the error function in Equation (1) and the design principles outlined in Equation (3), where $\mu_{1}\left(t\right)$ and $\mu_{2}\left(t\right)$ are defined as follows:

$$\begin{array}{c} \left\{\begin{array}{c} \mu_{1}\left(t\right)=3\exp\left(\frac{at}{2}\right)-\frac{a}{2},\\ \mu_{2}\left(t\right)=\exp\left(at\right). \end{array}\right. \end{array}$$

Here, t∈[0,+∞) and α∈(0,+∞). Notably, $\mu_{1}\left(t\right)$ and $\mu_{2}\left(t\right)$ are time-varying parameters that remain strictly positive. Additionally, D(t) denotes the external noise.

**Figure 4:**
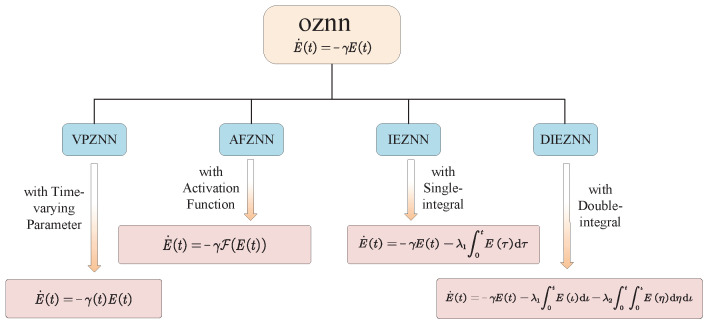
Taxonomy of ZNN Architectures.

Through rigorous theoretical analysis and proof, the superior performance of the VPNTZNN model in terms of convergence and robustness has been fully validated. For further developments on variable parameters, refer to Table 2. The taxonomy of ZNN architectures discussed in this section is illustrated in Figure 4.

## 4. Applications of Zeroing Neural Networks

This chapter will comprehensively discuss the research progress and practical applications of ZNN in various fields.

In 2023, Liao et al. proposed a dynamic robot position tracking method based on complex number representation (see reference [43]) and designed an optimization strategy for the real-time measurement and minimization of robot spacing. They further developed a CZND model for dynamic solving, with the formulation expressed as follows:

$$\begin{array}{c} E\Phi (t)+\Upsilon (t)=0. \end{array}$$

In the CTVME, Φ(t), representing the instantaneous position of the following robot, needs to be solved in real time. Based on the ZNN design framework, by solving the CTVME problem online, the zeroing neural dynamics method demonstrates its efficiency and feasibility in robot coordination. The error function is

$$\begin{array}{c} P(t)=E\Phi (t)+\Upsilon (t). \end{array}$$

This is used to quantify the error in the CTVME problem. The time derivative of P(t) is defined as follows:

$$\begin{array}{c} \dot{P}\left(t\right)=-\gamma \left(t\right)P\left(t\right). \end{array}$$

Further, we can obtain

$$\begin{array}{c} \dot{E}\Phi \left(t\right)+E\dot{\Phi }\left(t\right)+\dot{\Upsilon }\left(t\right)=-\gamma \left(E\Upsilon \left(t\right)+\Upsilon \left(t\right)\right). \end{array}$$

The CZND model is

$$\begin{array}{c} E\dot{\Phi }\left(t\right)=-\gamma E\Phi \left(t\right)-\dot{\Upsilon }\left(t\right)-\gamma \Upsilon \left(t\right). \end{array}$$

In 2014, Xiao et al. proposed a method based on the ZNN model to solve problems related to robotic arms [130]. The kinematic equation of the robotic arm is typically expressed as

$$\begin{array}{cc} \; & g(t)=f(\theta (t)),\\ \; & \dot{g}\left(t\right)=G\left(\theta \left(t\right)\right)\dot{\theta }\left(t\right). \end{array}$$

The error function is defined as follows:

$$\begin{array}{c} E\left(t\right)=r_{w}-r\left(t\right), \end{array}$$

where $r_{w}\left(t\right)$ represents the desired path to be tracked. By integrating the previously proposed formulas with the original ZNN model design equations outlined in the article, the wheeled mobile manipulator’s dynamics are derived as follows:

$$\begin{array}{c} G\left(\theta \left(t\right)\right)\dot{\theta }={\dot{r}}_{w}\left(t\right)+\gamma \left(r_{w}-f\left(\theta \left(t\right)\right)\right). \end{array}$$

A series of comprehensive experiments were carried out using the formulas outlined earlier. The results indicate that the ZNN method outperforms the traditional GNN approach in terms of accuracy.

With the development of ZNN, its application in robotic arm control has become increasingly widespread. Notable examples include minimum motion planning and control for redundant robotic arms [131,132], cooperative motion planning for robotic manipulator arms [83,84,93,106,133], multi-robot systems [134], path tracking for mobile robots [81,135], redundant robotic manipulators [86,87,136,137], four-joint planar robotic arms [47,94], motion tracking for mobile manipulators [102], coordinated path tracking for dual robotic manipulators [88], solving multi-robot tracking and formation problems [89], vehicular edge computing [138], and mobile object localization [49], among others.

Chaotic systems, first discovered by Edward Lorenz half a century ago, are a class of typical nonlinear systems [139]. Since their discovery, chaotic systems have become a focal point of research due to their wide range of practical applications, including in fields such as power systems [140], financial systems [141], ecological systems [142], and secure communication [143]. However, their inherent uncertainty, non-repeatability, and unpredictability make solving chaotic system problems highly challenging. The introduction of the ZNN model offers a reliable solution to effectively address issues in chaotic systems, particularly in environments with noise and uncertainties. The basic approach involves constructing models for the master and response systems:

$${\dot{x}}_{m}\left(t\right)=f_{m}\left(x_{m}\left(t\right)\right)+\omega \left(t\right),\;{\dot{x}}_{r}\left(t\right)=f_{r}\left(x_{r}\left(t\right)\right)+\mu \left(t\right),$$

where $x_{m}\left(t\right)$ and $x_{r}\left(t\right)$ represent the states of the master and response systems, $f_{m}\left(\cdot \right)$ and $f_{r}\left(\cdot \right)$ are the nonlinear dynamics of the systems, ω(t) denotes external disturbances, and μ(t) is the control input. The synchronization error is defined as $e\left(t\right)=x_{m}\left(t\right)-x_{r}\left(t\right)$, and the ZNN control law is given by $\dot{e}\left(t\right)=-\gamma e\left(t\right)$, ensuring exponential convergence of the error. To enhance noise immunity, the ZNN can incorporate integral and double-integral structures to mitigate low-frequency and high-frequency disturbances.

Studies have shown that ZNN-based models perform excellently in chaotic control. For example, Aoun et al. [144] proposed the NZNN, which successfully achieved three-dimensional synchronization in the SFM system. Xiao et al. [145] combined the ZNN with sliding mode control to develop the FXTRC strategy, achieving nearly 10 times faster convergence in various chaotic systems.

In 2023, Jin et al. introduced a time-varying fuzzy parameter zeroing neural network (TVFPZNN) model designed to achieve the synchronization of chaotic systems in the presence of external noise interference [146].

To demonstrate the superiority of the TVFPZNN, Jin conducted two synchronization experiments using the Chen chaotic system and an autonomous chaotic system, employing different fuzzy membership functions. During these experiments, three types of irregular noise were introduced to rigorously evaluate the model’s robustness. As documented in [146], the mathematical formulation of the Chen chaotic system is given by

$$\begin{array}{c} \left\{\begin{array}{c} {\dot{y}}_{1}\left(t\right)=a\left(y_{2}\left(t\right)-y_{1}\left(t\right)\right),\\ {\dot{y}}_{2}\left(t\right)=dy_{1}\left(t\right)-y_{1}\left(t\right)y_{3}\left(t\right)+cy_{2}\left(t\right),\\ {\dot{y}}_{3}\left(t\right)=y_{1}\left(t\right)y_{2}\left(t\right)-by_{3}\left(t\right). \end{array}\right. \end{array}$$

In the presence of external noise interference, the master chaotic system can be represented as

$$\begin{array}{c} \left\{\begin{array}{c} {\dot{y}}_{m1}\left(t\right)=a\left(y_{m2}\left(t\right)-y_{m1}\left(t\right)\right)+\varpi_{1}\left(t\right),\\ {\dot{y}}_{m2}\left(t\right)=dy_{m1}\left(t\right)-y_{m1}\left(t\right)y_{m3}\left(t\right)+cy_{m2}\left(t\right)+\varpi_{2}\left(t\right),\\ {\dot{y}}_{m3}\left(t\right)=y_{m1}\left(t\right)y_{m2}\left(t\right)-by_{m3}\left(t\right)+\varpi_{3}\left(t\right). \end{array}\right. \end{array}$$

The response chaotic system, incorporating the controller, can be represented as

$$\begin{array}{c} \left\{\begin{array}{c} {\dot{y}}_{r1}\left(t\right)=a\left(y_{r2}\left(t\right)-y_{r1}\left(t\right)\right)+\mu_{1}\left(t\right),\\ {\dot{y}}_{r2}\left(t\right)=dy_{r1}\left(t\right)-y_{r1}\left(t\right)y_{r3}\left(t\right)+cy_{r2}\left(t\right)+\mu_{2}\left(t\right),\\ {\dot{y}}_{r3}\left(t\right)=y_{r1}\left(t\right)y_{r2}\left(t\right)-by_{r3}\left(t\right)+\mu_{3}\left(t\right). \end{array}\right. \end{array}$$

As presented in reference [146], the mathematical formulation of the TVFPZNN model is given by

$$\begin{array}{cc} \; & f_{m}\left(z_{m}\left(t\right)\right)+\eta \left(t\right)-f_{r}\left(z_{r}\left(t\right)\right)-\mu \left(t\right)\\ \; & =-\left(a^{t+2k}+\lambda pt+p^{2}\right)F\left(z_{m}\left(t\right)-z_{r}\left(t\right)\right)+\eta \left(t\right). \end{array}$$

The expression $a^{t+2k}+\lambda pt+p^{2}$ denotes the fuzzy time-varying parameters.

In Experiment B [146], the researchers performed a comparative analysis of the PTVR-ZNN [147], AFT-ZNN [148], FPZNN [149], and TVFPZNN models for controlling the Chen chaotic system in both noise-free and noisy environments. The results demonstrated that, in the noise-free environment, all four models successfully achieved synchronization. However, in the presence of noise, only the TVFPZNN model was able to stably synchronize the Chen chaotic system. Moreover, under noise-free conditions, the Chen chaotic system controlled by the TVFPZNN exhibited the fastest convergence speed and the lowest error, further validating the superior performance of this model.

In Experiment C [146], the researchers examined the synchronization problem of the autonomous chaotic system. The mathematical formulation of the autonomous chaotic system is given by

$$\begin{array}{c} \left\{\begin{array}{c} {\dot{z}}_{1}\left(t\right)=p\left(z_{2}\left(t\right)-z_{1}\left(t\right)\right)+z_{2}\left(t\right)z_{3}\left(t\right),\\ {\dot{z}}_{2}\left(t\right)=\left(r-p\right)z_{1}\left(t\right)-z_{1}\left(t\right)z_{3}\left(t\right)+rz_{2}\left(t\right),\\ {\dot{z}}_{3}\left(t\right)=sz_{2}\left(t\right)z_{2}\left(t\right)-qz_{3}\left(t\right), \end{array}\right. \end{array}$$

Similarly, in the presence of external noise interference, the master chaotic system can be represented as

$$\begin{array}{c} \left\{\begin{array}{c} {\dot{z}}_{m1}\left(t\right)=p\left(z_{m2}\left(t\right)-z_{m1}\left(t\right)\right)+z_{m2}\left(t\right)z_{m3}\left(t\right)+\varpi_{1}\left(t\right),\\ {\dot{z}}_{m2}\left(t\right)=\left(r-p\right)z_{m1}\left(t\right)-z_{m1}\left(t\right)z_{m3}\left(t\right)+rz_{m2}\left(t\right)+\varpi_{2}\left(t\right),\\ {\dot{z}}_{m3}\left(t\right)=sz_{m2}\left(t\right)z_{m2}\left(t\right)-qz_{m3}\left(t\right)+\varpi_{3}\left(t\right). \end{array}\right. \end{array}$$

The response chaotic system, incorporating the controller, can be represented as

$$\begin{array}{c} \left\{\begin{array}{cc} {\dot{z}}_{r1}\left(t\right) & =p\left(z_{r2}\left(t\right)-z_{r1}\left(t\right)\right)+z_{r2}\left(t\right)z_{r3}\left(t\right)+\mu_{1}\left(t\right),\\ {\dot{z}}_{r2}\left(t\right) & =\left(r-p\right)z_{r1}\left(t\right)-z_{r1}\left(t\right)z_{r3}\left(t\right)+rz_{r2}\left(t\right)+\mu_{2}\left(t\right),\\ {\dot{z}}_{r3}\left(t\right) & =sz_{r2}\left(t\right)z_{r2}\left(t\right)-qz_{r3}\left(t\right)+\mu_{3}\left(t\right). \end{array}\right. \end{array}$$

In this context, π(t) and μ(t) represent the controllers.

The experiment evaluated the performance of the aforementioned models in controlling the autonomous chaotic system under noisy conditions. The results showed that the TVFP-ZNN model outperformed the other models by a significant margin.

Over the past decade, the rapid development of the ZNN has made a significant impact across various fields. It has demonstrated significant effectiveness, particularly in robotic control. In tasks such as trajectory tracking, motion planning, and formation control, the ZNN is especially suitable for real-time control applications due to its rapid convergence and ability to handle disturbances, providing precise responses. Table 3 presents a performance comparison of the ZNN across various applications. Additionally, the ZNN has been successfully applied in chaos system control, drone coordination, and chaos circuit synchronization, highlighting its versatility and strong performance in dynamic control tasks. In addition to its applications in robot control and chaotic systems, as mentioned earlier, the ZNN has also been widely used in image information processing [150,151], multidimensional spectral estimation [152], mathematical ecology [153], IPC system pendulum tracking [154], and mobile target localization [155,156,157], among other areas.

## 5. Conclusions

This paper presents a comprehensive review on the application of the ZNN model in addressing time-varying problems, focusing on its model structure. The models discussed include single-integral and double-integral structures with noise immunity, general nonlinear function structures, finite-time convergence structures, fixed-time convergence structures, predefined-time convergence structures, and variable-parameter structures. Furthermore, the paper also explores the robustness of the ZNN in addressing noise, external disturbances, and system uncertainties, demonstrating its engineering practicality in tasks such as trajectory tracking and chaos control. The successful application of the ZNN in complex systems, such as multi-arm collaborative control, multi-agent formation, and nonholonomic robot path planning, highlights its powerful capability in handling high-dimensional, dynamic, and coupled problems.

As the ZNN model continues to evolve, its applications have become widespread across various practical domains. However, several key challenges still persist in this field. (1) Higher-order dynamics or multiple-integral structures improve performance but increase computational complexity. In real-time or resource-constrained environments, balancing performance and computational cost is a key challenge. (2) The ZNN model relies on gradient information, making it suitable for convex optimization problems, but it still faces challenges in non-convex or multi-modal optimization problems. In the future, combining the ZNN with swarm intelligence or evolutionary algorithms could enhance its global search capability. (3) The application of zeroing neural networks could be expanded to more fields. In conclusion, this review provides a reference for beginners who wish to gain a deeper understanding of how zeroing neural networks efficiently solve time-varying problems.

## Figures and Tables

**Table 1:** Activation functions and their convergence.

Activation Function	Parameter	Convergence
$\frac{1}{2}({\left|e\right|}^{\kappa }+{\left|e\right|}^{\frac{1}{\kappa }})\mathrm{sgn}\left(e\right)$	κ>0	Finite-time [109]
$\left\{\begin{array}{cc} \frac{b}{a}\sqrt{2a\;|e_{ij}\left|-\right|e_{ij}{|}^{2}}, & |e_{ij}|<a\\ b, & |e_{ij}|\geq a \end{array}\right.$	a,b>0	Finite-time [110]
$\left\{\begin{array}{c} \frac{1-\exp(-\varepsilon x)}{1+\exp(-\varepsilon x)}\cdot \frac{1+\exp(-\varepsilon )}{1-\exp(-\varepsilon )} \end{array}\right.$	ε>0	Finite-time [111]
$(\rho_{1}|x{|}^{a}+\rho_{2}|x{|}^{1/a}+\rho_{3}|x|)\mathrm{sign}\left(x\right)$	$\rho_{1},\rho_{2},\rho_{3},\rho_{4},a>0$	Finite-time [75]
$(b_{1}{\left|e\right|}^{\kappa }+b_{2}{\left|e\right|}^{\eta })\mathrm{sgn}\left(e\right)+b_{3}e$	$b_{1},b_{2},b_{3},\kappa ,\eta >0$	Fixed-time [112,113]
$\left(a_{1}{\left|x\right|}^{\nu }+a_{2}{\left|x\right|}^{\nu +1}+a_{3}\left|x\right|-a_{4}\right)\mathrm{sgn}\left(x\right)$	$a_{1},a_{2},a_{3},a_{4},\nu >0$	Fixed-time [80]
$\frac{1}{p}\left(\kappa_{1}{\left|x\right|}^{1-p}+\kappa_{2}{\left|x\right|}^{q}\right)\mathrm{sign}\left(x\right)+\kappa_{3}\mathrm{sign}\left(x\right)$	$q,\kappa_{1},\kappa_{2},\kappa_{3}>0,p\in \left(0,1\right)$	Fixed-time [114]
$h\sin(|x{|}^{n})\mathrm{sign}\left(x\right)$	h,n>0	Fixed-time [115]
$\left\{\begin{array}{cc} \frac{e}{t_{m}-t}, & t\in [0,t_{m})\\ e+{\left|e\right|}^{q}\mathrm{sgn}\left(e\right)+p\mathrm{sgn}\left(e\right), & t\in [t_{m},+\infty ) \end{array}\right.$	$t_{m},q,p>0$	Predefined-time [105]
$\left\{\begin{array}{cc} \frac{\exp(e)-1}{\exp\left(e\right)\left(t_{m}-t\right)}, & t\in [0,t_{m})\\ e, & t\in [t_{m},+\infty ) \end{array}\right.$	$t_{m}>0$	Predefined-time [116]
$(d_{1}{\left|e\right|}^{\nu }+d_{2}{\left|e\right|}^{\epsilon })\mathrm{sgn}\left(e\right)+d_{3}\mathrm{sgn}\left(e\right)+d_{4}e$	$d_{1},d_{2},d_{3},d_{4},\nu ,\epsilon >0$	Predefined-time [86,117]
$\left(c_{1}\sum_{i=1}^{m}{\left|e\right|}^{\nu_{i}}+c_{2}\sum_{i=1}^{m}{\left|e\right|}^{\kappa_{i}}\right)\mathrm{sgn}\left(e\right)+c_{3}e+c_{4}\sinh\left(e\right)$	$c_{1},c_{2},c_{3},c_{4},\nu_{i},\kappa_{i}>0$	Predefined-time [118]
$\frac{\eta }{c}{\exp(|x|}^{c}{)|x|}^{1-c}\mathrm{sign}\left(x\right)+\nu \mathrm{sign}\left(x\right)$	η,ν>0,c∈(0,1)	Predefined-time [119]

**Table 2:** Development of varying parameters.

Variable Parameter	Parameter	Year	Literature
$\theta \left(t\right)=\zeta +t^{\gamma }$	ζ>0,γ>0	2018	[121]
$\theta \left(t\right)=p^{t}+p$	p>0	2019	[122]
$\theta \left(t\right)=t^{p}+p$	p>0	2019	[123]
$\dot{\theta }\left(t,x\right)=c\cdot \mathrm{sign}\left(|x|\right)$	c>0	2020	[124]
$\theta \left(t\right)=\left\{\begin{array}{cc} t^{q}+q, & \mathrm{if}\;0<q\leq 1\\ q^{t}+2qt+p, & \mathrm{if}\;q>1 \end{array}\right.$	q>0	2021	[125]
$\theta \left(t\right)=\beta \exp(\lambda_{1}\mathrm{arccot}\left(t\right)+\lambda_{2}t)$	$\beta ,\lambda_{1},\lambda_{2}>0$	2021	[126]
$\left\{\begin{array}{c} \theta_{1}\left(t\right)=3\exp\left(\frac{\alpha t}{2}\right)-\frac{\alpha }{2}\\ \theta_{2}\left(t\right)=\exp\left(\alpha t\right) \end{array}\right.$	α>0	2022	[120]
$\theta \left(t\right)=\left\{\begin{array}{cc} \gamma_{1}k_{1}^{\alpha_{1}t}, & \mathrm{if}\;t<\delta_{0}\\ \gamma_{1}k_{1}^{\alpha_{1}\delta_{0}}, & \mathrm{if}\;t\geq \delta_{0} \end{array}\right.$	$\gamma_{1},k_{1},\alpha_{1},\delta_{0}>0$	2023	[96]
θ(t)=γexp(∥E(t)∥)	γ>0	2023	[127]
$\theta \left(t\right)=\varrho \exp\left(\left(t^{\kappa }+\kappa \right)\vartheta \left(t\right)\right)$	ϱ,κ>0	2024	[128]
$\theta \left(t\right)=\varrho \exp(\left(\beta^{t}+\beta \right)∥E\left(t\right)∥)$	ϱ,β>0	2024	[129]

**Table 3:** Performance comparison table of ZNN in various applications.

Model	Application Scenarios	Position Error	Integral Structure	Reference
HADTZTM	Manipulator motion planning	$10^{-5}$	No	[158]
FTZNN	Manipulator motion planning	$10^{-5}$	No	[159]
ITFCZNN	Manipulator motion planning	$10^{-4}$	No	[160]
RZND	Manipulator motion planning	$10^{-4}$	Single	[161]
FTCND	Manipulator motion planning	$10^{-4}$	No	[162]
STZNN	Manipulator motion planning	$10^{-5}$	Single	[163]
VP-CDNN	Manipulator motion planning	$10^{-7}$	No	[121]
DZNN	Manipulator motion planning	$10^{-8}$	No	[164]
CNDSM	Manipulator motion planning	$10^{-4}$	Single	[165]
FER-DZNN	Manipulator motion planning	$10^{-5}$	Single	[166]
CZND	multi-agent system control	$10^{-4}$	No	[43]
AP-FTZND	multi-agent system control	$10^{-7}$	No	[167]
TVFP-ZNN	Chaotic system	$10^{-6}$	No	[146]
NZNN	Chaotic system	$10^{-4}$	Single	[144]

## Data Availability

Not applicable.

## Abbreviations

The following abbreviations are used in this manuscript:

ZNN	zeroing neural network
GNN	gradient neural network
TVCMI	time-varying complex matrix inversion
TVCMP	time-varying complex matrix pseudoinversion
TVNE	time-varying nonlinear equation
TVOLS	time-varying overdetermined linear system
TVSME	time-varying Stein matrix equation
TVNM	time-varying nonlinear minimization
NNP	nonconvex nonlinear programming
MOO	multi-objective optimization
TVQO	time-varying quadratic optimization
TVP	time-varying problems
NT	noise-tolerant
RNN	recurrent neural network
VEH	harris hawks algorithm
ZND	zeroing neural dynamics
BZND	bounded zeroing neural dynamics
NCZNN	novel complex-valued zeroing neural network
NIEZNN	nonlinear activation integral-enhanced zeroing neural network
C-AF	coalescent activation function
FTNTZNN	fixed-time noise-tolerant zeroing neural network
VAF	versatile activation function
NRNN	novel recursive neural network
TVFPZNN	time-varying fuzzy parameter zeroing neural network
VPNTZNN	variable-parameter noise-tolerant zeroing neural network
FT-VP-CDNN	finite-time varying-parameter convergent differential neural network
VP-CDNN	variable-parameter recurrent neural network
